# Supplementary material for: Graded decisions in the human brain
Source: Nat Commun. 2024 May 21;15:4308. doi: 10.1038/s41467-024-48342-w (PMC11109249; doi:10.1038/s41467-024-48342-w)
Supplement: Supplementary file 1 — Supplementary Information [file 41467_2024_48342_MOESM1_ESM.pdf]

Supplementary Information

Graded decisions in the human brain

Tao Xie[1,2], Markus Adamek[1,2], Hohyun Cho[1,2], Matthew A. Adamo[3], Anthony L. Ritaccio[4,5], Jon T. Willie[1,2], Peter Brunner*[1,2,4], Jan Kubanek*[6]

[1]Department of Neurological Surgery, Washington University School of Medicine, St. Louis, MO 63110, USA

[2]National Center for Adaptive Neurotechnologies, St. Louis, MO 63110, USA

[3]Department of Neurosurgery, Albany Medical College, Albany, NY 12208, USA

[4]Department of Neurology, Albany Medical College, Albany, NY 12208, USA

[5]Department of Neurology, Mayo Clinic, Jacksonville, FL 32224, USA

[6]Department of Biomedical Engineering, University of Utah, Salt Lake City, UT 84112, USA

* Corresponding authors: pbrunner@wustl.edu, jan.kubanek@utah.edu

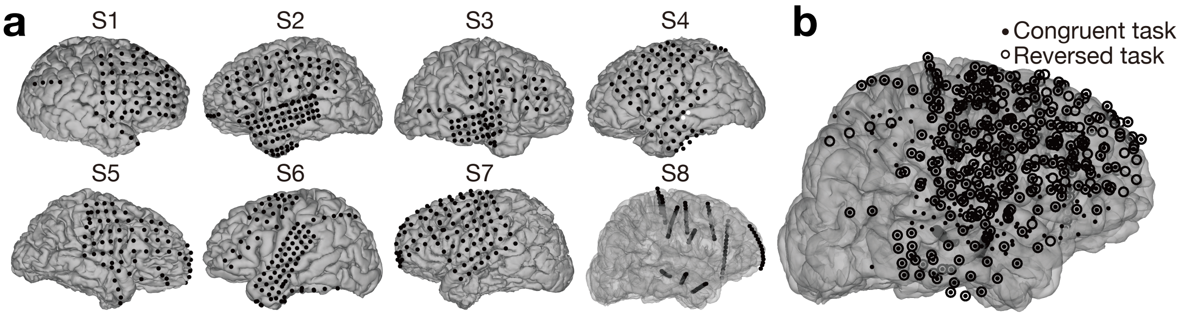

c

Subject ID	Handed-ness	Sex	Implanted hemisphere	Number of electrodes	Number of performed trials	
					Congruent task	*Reversed task*
S1	R	F	R	66 (71)	N/A	(346,359,110)
S2	R	M	L	89 (134)	(111,154,60)	N/A
S3	L	M	R	46 (76)	(289,306,79)	(208,245,66)
S4	L	M	L	69 (81)	(87,90,29)	(84,178,37)
S5	R	F	R	45 (85)	(271,279,68)	(259,251,47)
S6	R	F	L	70 (98)	(105,252,56)	N/A
S7	R	M	L	89 (126)	(173,169,50)	(123,252,57)
S8	R	M	R	97 (128)	(490,434,191)	(211,227,84)

R: right; L: left; F: female; M: male

Number of electrodes: Included in analyses (Total). Number of performed trials: (Saccade choice, Button press choice, Incorrect choice)

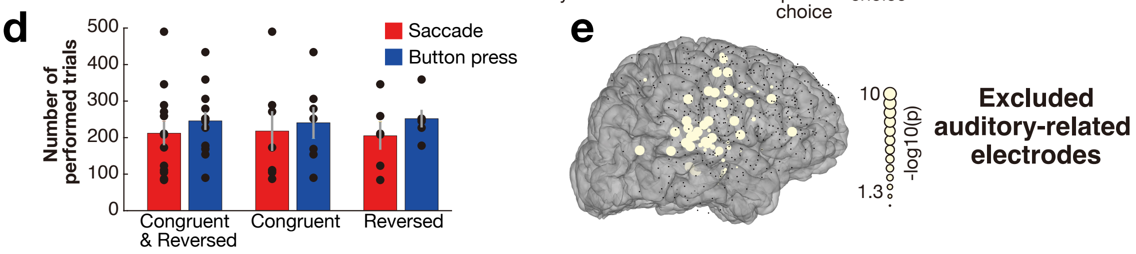

Figure S1. **Subjects information.** **a**, Electrode coverage for all subjects. The black dots show the locations of all 799 electrodes (prior visual inspection) inferred from CT scans. **b**, Electrode locations were pooled across the subjects and projected onto a template brain. For visualization purposes, we projected the left hemisphere electrodes of subjects S2, S4, S6, and S7 onto the right hemisphere. Solid/hollow circles indicate the location of all 571 electrodes (after visual inspection) across all subjects engaged in the congruent/reversed tasks, respectively. **c**, Information of individual subjects. 8 humans (with mean±s.d. age and IQ equal to 39±15 and 94.5±17.2, respectively) with intractable epilepsy participated in our study. **d** Number (mean±s.e.m.) of performed trials in congruent and reversed tasks. The black circles indicate the average number of trials for each session. Specifically, subjects performed an average of 218 (241) congruent trials for the saccade (button press) choice, while subjects performed an average of 205 (252) reversed trials. **e**, Auditory-related electrodes that were excluded. Electrodes with significant ($p < 0.05$, corrected using false discovery rate, one-tailed randomization tests) broadband gamma increase for both the decision task and passive listening task were defined as auditory-related electrodes. We extracted 79/571 auditory-related electrodes from eight subjects, and these electrodes were excluded. Together, 492 electrodes in eight subjects were included for further analyses.

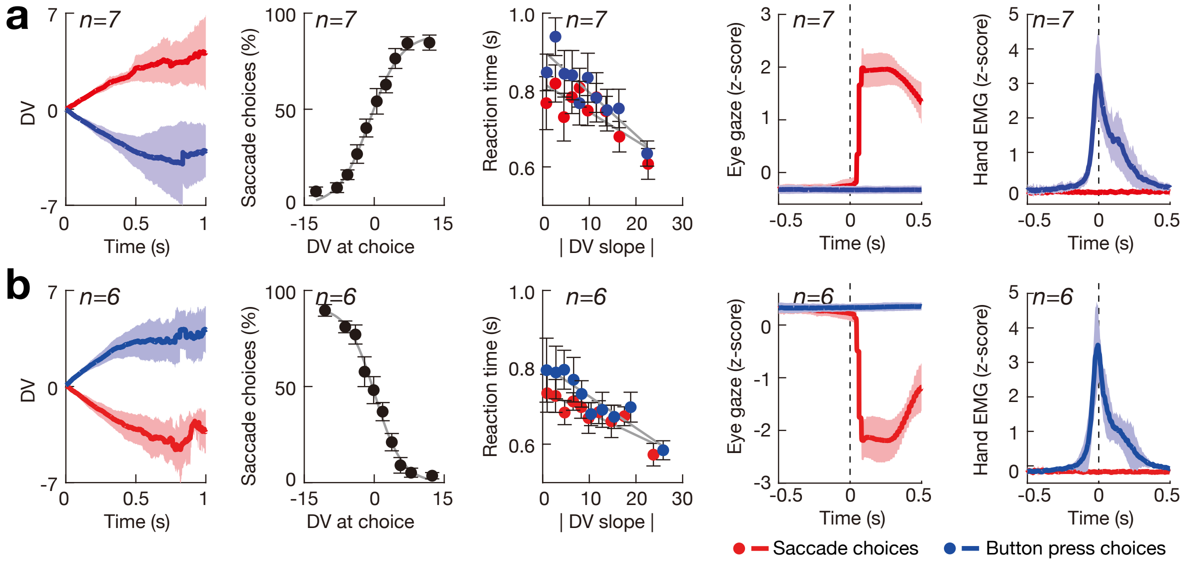

Figure S2. **Choice behavior in the two task contexts.** **a**, Congruent task context ($n = 7$ sessions). **b**, Reversed task context ($n = 6$ sessions). Panels from left to right: Mean±s.d. DV during the stimulus interval; Mean±s.e.m. proportion of saccade choices as a function of the DV at the time of choice (100 ms preceding movement onset); Mean±s.e.m. reaction time as a function of the absolute value of the DV slope; Mean±s.d. eye gaze across sessions, aligned to saccade onset; Mean±s.d. electromyographic activity (EMG, recorded from the hand performing the button press) across sessions, aligned to button press onset. The data are shown separately for trials that resulted in a saccade (red) and trials that resulted in a button press (blue).

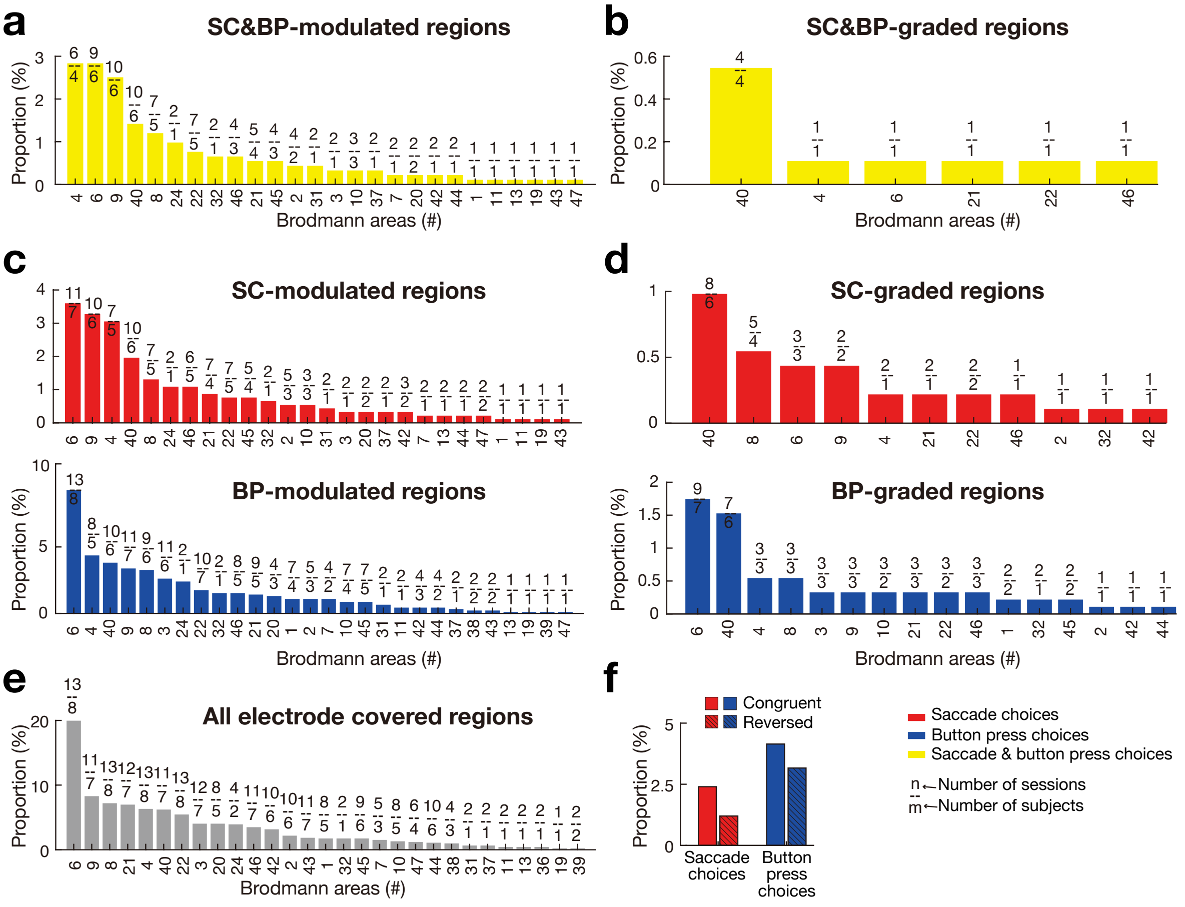

Figure S3. **Effector-modulated and DV-graded Brodmann areas.** **a**, SC&BP-modulated electrodes (**Fig. 3a**), which show significant γ modulation for both saccade and button press choices. **b**, SC&BP-graded electrodes (**Fig. 5a**), in which the γ signals were significantly modulated by the DV at the time of choice for both saccade and button press choices. **c**, SC-modulated and BP-modulated electrodes (**Fig. 3b**), which show significant γ modulation for saccade and button press choices, respectively. **d**, SC-graded and BP-graded electrodes (**Fig. 5b**), in which the γ signals were significantly modulated by the DV at the time of choice for saccade and button press choices, respectively. **e**, The proportion of electrodes located within individual Brodmann areas. **f**, DV-graded electrodes (in **d**) during congruent and reversed task contexts. Specifically, the proportion of SC-graded and BP-graded electrodes in congruent (reversed) sessions was 2.4% (1.2%) and 4.1% (3.2%), respectively. Subject S8 also provided access to deep brain targets. Electrodes from all 13 sessions (8 subjects) are included. The numbers above each bar indicate the number of sessions (numerator) and number of subjects (denominator).

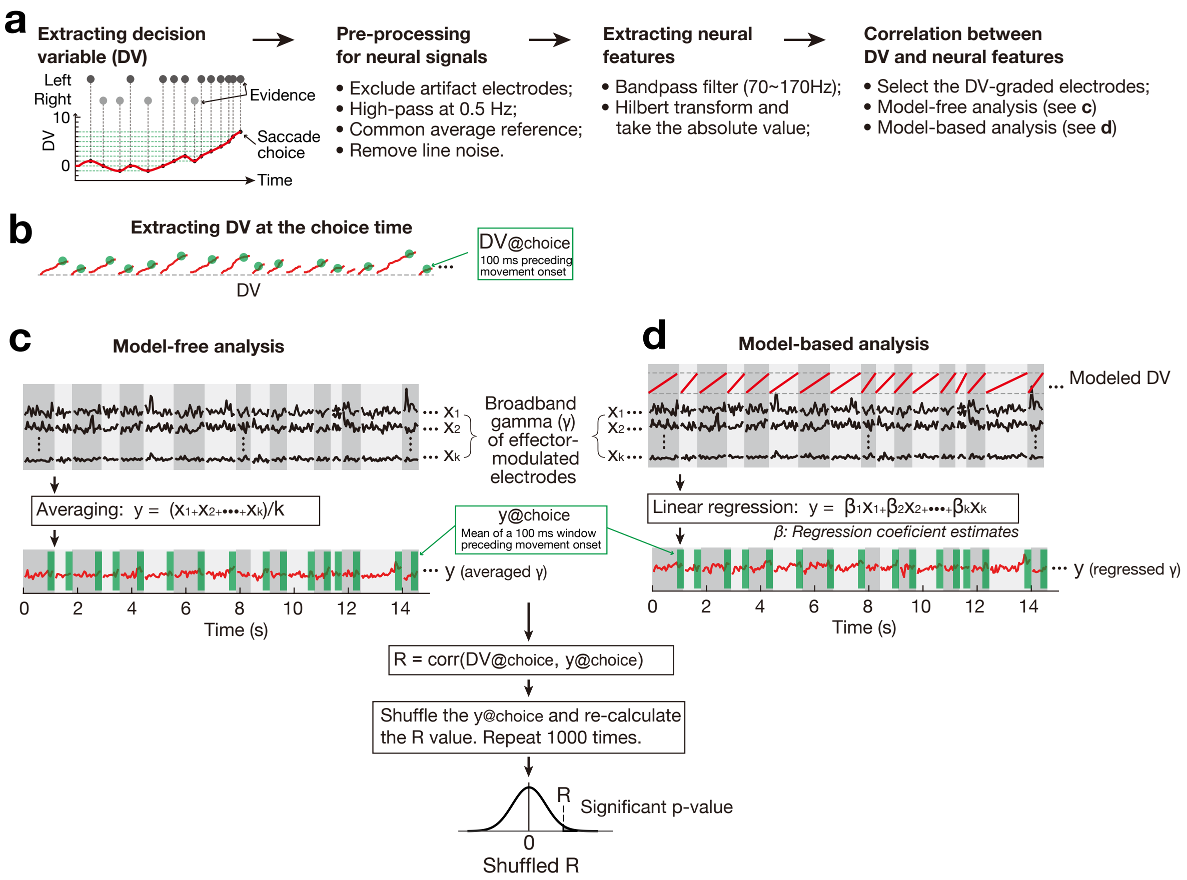

Figure S4. **A graphical rendering of the analyses.** **a**, Signal processing. The decision variable (DV, red trace) was derived from the presented evidence (black dots indicating time and laterally of click sounds) in the same way as many previous studies[15,70]. **b**, DV at the time of choice (DV@choice). We define the DV at the time of choice as the DV value 100 ms preceding movement onset. **c**, Model-free analysis. We averaged the broadband gamma (γ) from effector-modulated electrodes (black traces), and calculated the mean value of the averaged γ over a 100 ms window preceding movement onset for each trial (y@choice). **d**, Model-based analysis. We applied a linear regression between the γ of the effector-modulated electrodes (black traces) and the corresponding value of the modeled DV (red trace). This provided a set of weights that enabled us to predict the modeled DV from the γ. Next, we computed the correlation between y@choice and DV@choice, and determined its significance using a randomization test. To avoid the potential mixing of decision difficulty and response type, we have separated saccade and button press trials in the model-free and model-based analysis. The analysis of the congruent and reversed data was also performed separately. We used saccade trials as an example in this graphical figure.

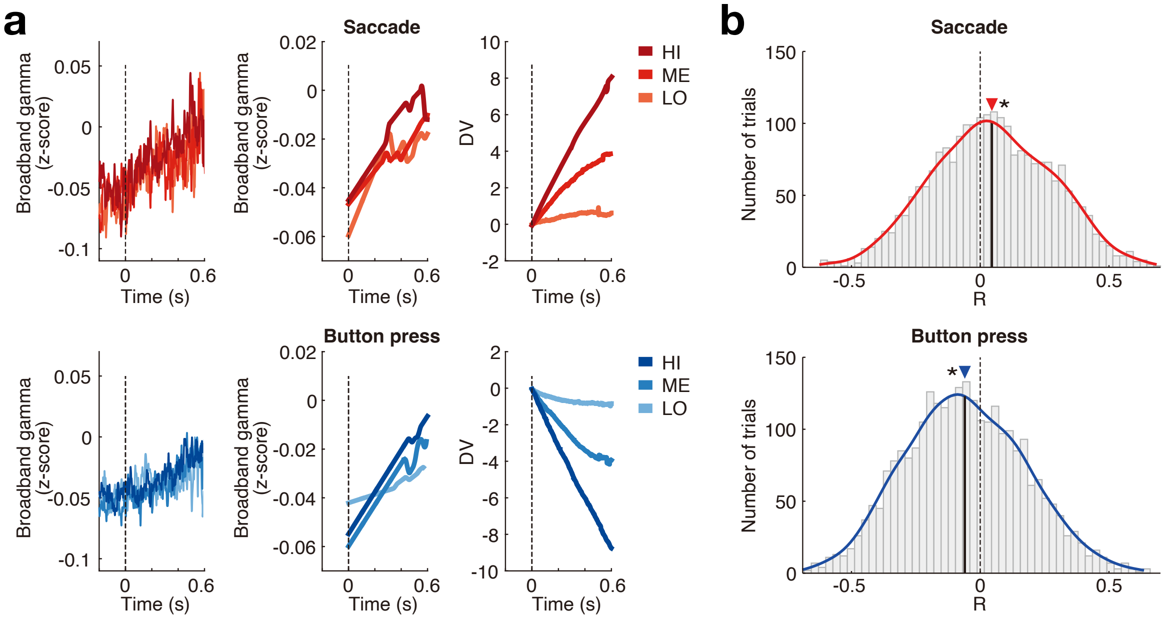

Figure S5. **Effector-related broadband gamma (γ) signals index developing decisions. a**, Decision signals in effector-modulated regions. Left panel: Mean ($n = 13$ sessions) γ activity of SC-modulated (top panel) and BP-modulated (bottom panel) regions (**Fig. 3b**) for the three different levels of decision evidence (HI, ME, LO). Middle panel: Mean ($n = 13$) of least squares fitted γ activity of SC-modulated (top panel) and BP-modulated (bottom panel) regions. Right panel: Mean ($n = 13$) value of the DV as a function of time. The signals are aligned to the stimulus onset (dashed line). The top (bottom) panel shows γ activity (left, middle panels) and DV (right panel) for trials that resulted in a saccade (button press) choice. **b**, Spearman's correlation R between the time course of γ activity and the time course of DV, throughout each decision period (stimulus onset to the time of choices). The individual values in the histogram represent individual trials, and are presented separately for saccade (top histogram) and button press (bottom histogram) choices. The triangle denotes the mean R value. $*$: $p = 8.4 \times 10^{-21}$ and $p = 8.1 \times 10^{-40}$ for saccade and button press, respectively (two-tailed t-tests).

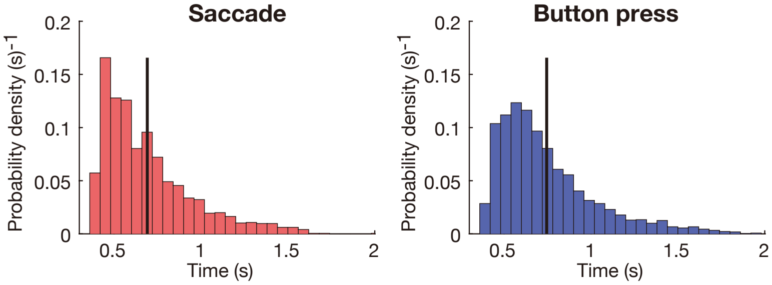

Figure S6. **Distribution of reaction time.** Reaction time distributions for saccade (left panel) and button press (right panel) choices across all trials. The vertical black line indicates the mean reaction time.

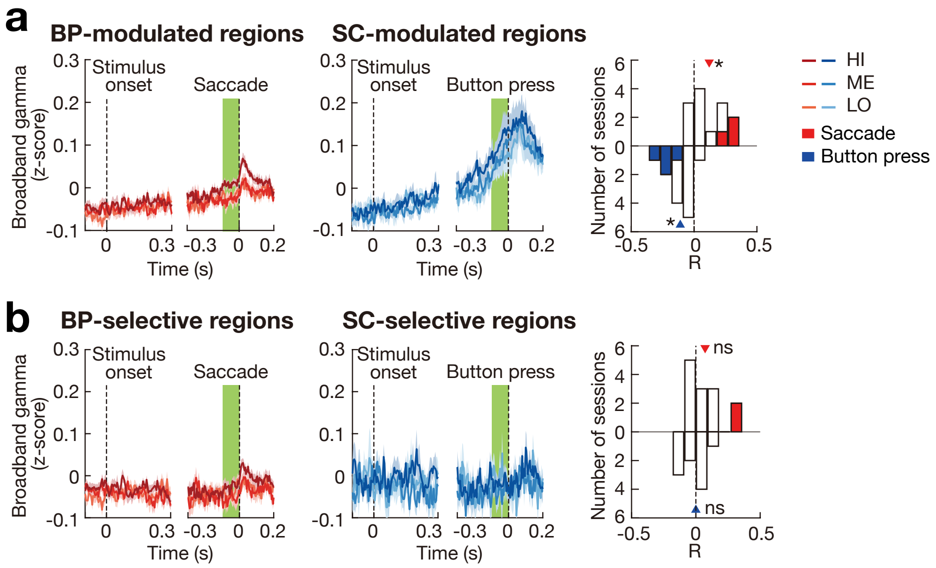

Figure S7. **Effects for opposite choice kind in effector-modulated regions and effector-selective regions. a** Graded effect in effector-modulated regions. Left panel: Session-mean ($\pm$ s.e.m., $n = 13$) broadband gamma (γ) activity of button press (BP)-modulated regions for trials that resulted in saccade choices. Middle panel: Session-mean ($\pm$ s.e.m., $n = 13$) γ activity of saccade (SC)-modulated regions for trials that resulted in button press choices. Right panel: Spearman's correlation R between trial-by-trial values of reaction time and γ activity around the time of choice (green bars). Bars in the histogram represent individual sessions ($n = 13$) and are presented separately for saccade (top histogram) and button press (bottom histogram) choices. The color-filled bars denote significant R values ($p < 0.01$, one-tailed randomization tests). $*$: BP-modulated regions were significantly graded by the DV at the time of saccade choices (top histogram) with an average $R = 0.12$ ($t(12) = 3.4$, $p = 5.6 \times 10^{-3}$, two-tailed t-test); SC-modulated regions were significantly graded by the DV at the time of button press choices (bottom histogram) with an average $R = -0.11$ ($t(12) = -3.9$, $p = 2.0 \times 10^{-3}$, two-tailed t-test). **b** Graded effect in effector-selective regions. Effector-selective regions were defined as those exclusively modulated by the effector indicated in the subtitle. Same analyses and format as **a**, but for the BP-selective and SC-selective electrodes. The BP-selective regions were not graded (ns, $p > 0.05$) by the DV at the time of saccade choices (top histogram) with an average $R = 0.07$ ($t(12) = 2.0$, $p = 0.07$; two-tailed t-test). Similarly, The SC-selective regions were not graded by the DV at the time of button press choices (bottom histogram) with an average $R = -0.0$ ($t(9) = -0.0$, $p = 1.0$; two-tailed t-test). We used the same format and analysis as in **Fig. 3b**.

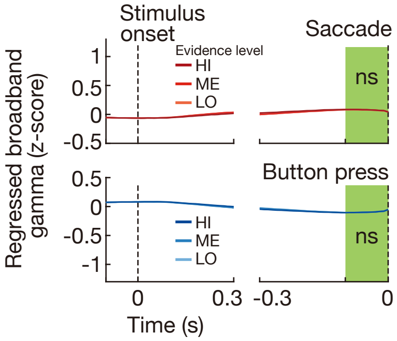

Figure S8. **Randomization analysis.** Same as **Fig. 4b** with the broadband gamma signals circularly shuffled (mean $\pm$ s.e.m., $n = 13$). ns: $F(2, 36) = 5.11$, $p = 0.011$ for saccade; $F(2, 36) = 0.40$, $p = 0.68$ for button press; one-way ANOVA.

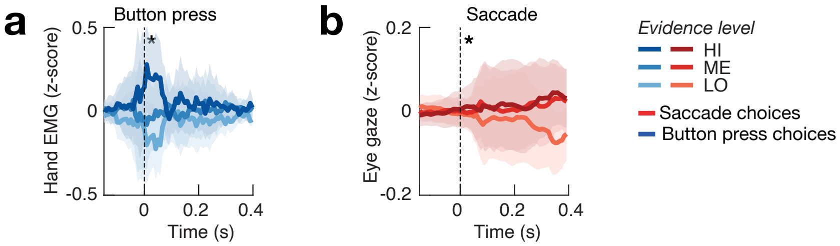

Figure S9. **Decision-related signals in the peripheral nervous system.** **a**, Electromyographic (EMG) activity of the hand pressing the button (mean $\pm$ s.e.m., $n = 13$) as a function of time. **b**, Horizontal eye gaze (mean $\pm$ s.e.m., $n = 13$) as a function of time. To better visualize the DV-graded effect, in each session, the EMG and eye gaze signals were normalized by subtracting the trial-averaged signal for saccade and button press choice, respectively. We explored the DV-graded effect in the effector peripheral systems that execute each choice. On each trial, the EMG amplitude was averaged between 50 ms preceding and 50 ms following a button press. The amplitude of eye gaze was taken as the maximal value within the period from 200 ms preceding to 200 ms following saccade onset. We found that for valid trials, hand EMG and eye gaze were significantly ($p < 0.05$) graded by the DV at the time of choice using the same analysis as **Fig. 3b**. $*$: $t(12) = 4.0$, $p = 0.0017$ for hand EMG in button press choices; $t(12) = 3.1$, $p = 0.010$ for eye gaze in saccade choices; two-tailed t-tests.
