# Supplementary material for: Graded decisions in the human brain
Source: Nat Commun. 2024 May 21;15:4308. doi: 10.1038/s41467-024-48342-w (PMC11109249; doi:10.1038/s41467-024-48342-w)
Supplement: Supplementary file 3 — Reporting Summary [file 41467_2024_48342_MOESM3_ESM.pdf]

Corresponding author(s): Peter Brunner, Jan Kubanek

Last updated by author(s): Mar 15, 2024

Reporting Summary

Nature Portfolio wishes to improve the reproducibility of the work that we publish. This form provides structure for consistency and transparency in reporting. For further information on Nature Portfolio policies, see our Editorial Policies and the Editorial Policy Checklist.

Statistics

For all statistical analyses, confirm that the following items are present in the figure legend, table legend, main text, or Methods section.

n/a	Confirmed	
☐	☒	The exact sample size (*n*) for each experimental group/condition, given as a discrete number and unit of measurement
☐	☒	A statement on whether measurements were taken from distinct samples or whether the same sample was measured repeatedly
☐	☒	The statistical test(s) used AND whether they are one- or two-sided<br>*Only common tests should be described solely by name; describe more complex techniques in the Methods section.*
☐	☒	A description of all covariates tested
☐	☒	A description of any assumptions or corrections, such as tests of normality and adjustment for multiple comparisons
☐	☒	A full description of the statistical parameters including central tendency (e.g. means) or other basic estimates (e.g. regression coefficient) AND variation (e.g. standard deviation) or associated estimates of uncertainty (e.g. confidence intervals)
☐	☒	For null hypothesis testing, the test statistic (e.g. *F*, *t*, *r*) with confidence intervals, effect sizes, degrees of freedom and *P* value noted<br>*Give P values as exact values whenever suitable.*
☒	☐	For Bayesian analysis, information on the choice of priors and Markov chain Monte Carlo settings
☒	☐	For hierarchical and complex designs, identification of the appropriate level for tests and full reporting of outcomes
☐	☒	Estimates of effect sizes (e.g. Cohen's *d*, Pearson's *r*), indicating how they were calculated

Our web collection on statistics for biologists contains articles on many of the points above.

Software and code

Policy information about availability of computer code

Data collection	The eye gaze position of each eye was measured 60 times/second using an eye tracker (Tobii T60, Tobii Technology) integrated into the flat-screen monitor. The hand contralateral to the recording hemisphere rested on a pillow placed on a fixed table while holding a joystick (Logitech Attack 3). Intracranial neural signals and EMG (pre-gelled disposable Ag/AgCl sEMG electrodes) signals were simultaneously amplified and sampled at 1200 Hz in a manner that prevents aliasing (g.USBamp/g.Hlamp biosignal acquisition devices, g.tec). Synchronized acquisition of neural signals, EMG signals, eye gaze, joystick responses, and task control was accomplished with BCI2000.
Data analysis	We established the anatomical locations of the implanted electrodes by co-registering the post-operative computer tomography image (CT) with a pre-operative magnet resonance imaging (MRI) T1 image. We used FreeSurfer (version 7.2.0) to generate a 3D model of the cortical surface. We used Hamming-windowed sinc FIR filters for all the filtering processes (EEGLAB, version 2022.0). All further signal analyses are performed in Matlab (version 2017b, Mathworks, Inc., Natick, MA).

For manuscripts utilizing custom algorithms or software that are central to the research but not yet described in published literature, software must be made available to editors and reviewers. We strongly encourage code deposition in a community repository (e.g. GitHub). See the Nature Portfolio guidelines for submitting code & software for further information.

Data

Policy information about availability of data

All manuscripts must include a data availability statement. This statement should provide the following information, where applicable:

- Accession codes, unique identifiers, or web links for publicly available datasets
- A description of any restrictions on data availability
- For clinical datasets or third party data, please ensure that the statement adheres to our policy

Source data are provided with this paper. Full datasets from our clinical recordings will be provided to interested researchers upon reasonable request to the corresponding authors and institutional approval of a data-sharing agreement.

Research involving human participants, their data, or biological material

Policy information about studies with human participants or human data. See also policy information about sex, gender (identity/presentation), and sexual orientation and race, ethnicity and racism.

Reporting on sex and gender	8 humans (5 males, 3 females) with drug-resistant epilepsy participated in our study. The sex of all participants is reported. Sex was not factored into study design.
Reporting on race, ethnicity, or other socially relevant groupings	In our study, we did not group participants based on race, ethnicity, or other socially relevant groupings.
Population characteristics	8 humans (5 males, 3 females, 6 right-handed, 2 left-handed, aged 15 to 57 (mean±s.d. age of 39±15), recorded from July/2012 to February/2020) participated in our study. The subjects underwent surgery for temporary placement of subdural grid electrodes (7 subjects) or intracerebral stereotactical electrodes (1 subject) to localize their epileptogenic focus. All subjects had normal cognitive capacity (mean±s.d. IQ of 94.5±17.2), normal hearing, normal or corrected-to-normal vision, and were able to perform the highly controlled task.
Recruitment	Subjects with an IQ of 70 or higher, implanted with intracranial electrodes over hand motor cortex and auditory cortex were referred by treating physicians at Albany Medical College. Informed consent was obtained prior participating in the study. All subjects were informed that their decision to participate in the research study was strictly voluntary, and would not impact their clinical treatment. We did not expect self-selection biases in our study.
Ethics oversight	The study was approved by the Institutional Review Board of Albany Medical College. All subjects gave informed consent to participate in the study. For subjects below the age of 18 years, informed consent was obtained from their legal guardians.

Note that full information on the approval of the study protocol must also be provided in the manuscript.

Field-specific reporting

Please select the one below that is the best fit for your research. If you are not sure, read the appropriate sections before making your selection.

☒ Life sciences ☐ Behavioural & social sciences ☐ Ecological, evolutionary & environmental sciences

For a reference copy of the document with all sections, see nature.com/documents/nr-reporting-summary-flat.pdf

Life sciences study design

All studies must disclose on these points even when the disclosure is negative.

Sample size	The population consisted of 8 subjects with drug-resistant epilepsy. No sample size calculation was performed. This sample size is enough for our work as our study performed the analyses for each individual separately. The time length of the neural recordings collected for each subject was based on surgical considerations as well as the amount of time each subject was willing to volunteer for the study. The data collected from each subject were sufficient to perform the statistic analyses.
Data exclusions	If subjects broke fixation for more than 150 ms, pressed any button before the auditory stimulus onset, responded with both button press and eye movements, or failed to indicate a response within 2s following the stimulus onset, the trial was aborted and excluded from analyses. The incorrect response trials were further excluded to eliminate the potential confound related to error response. Additionally, two subjects started participating in the experiment, but were not able to complete the minimum amount of required trials due to having frequent seizures. Therefore, we excluded the two subjects from the analyses.
Replication	Our analysis was performed for each individual separately rather than on a group level. We test the decision-related graded effect for each individual, and found a significant graded effect across individuals. Therefore, our results demonstrate the reproducibility of our experimental findings.
Randomization	There was no group allocation in this study. We used a within-subject design and as such randomization across subjects was not needed.

Blinding	There was no group allocation in this study, therefore blinding to group allocation was not relevant to this study. Additionally, blinding was not necessary as the experimenters' knowledge of participant assignment during data collection and analysis do not influence the outcome of the results.

Reporting for specific materials, systems and methods

We require information from authors about some types of materials, experimental systems and methods used in many studies. Here, indicate whether each material, system or method listed is relevant to your study. If you are not sure if a list item applies to your research, read the appropriate section before selecting a response.

Materials & experimental systems

n/a	Involved in the study
☒	☐ Antibodies
☒	☐ Eukaryotic cell lines
☒	☐ Palaeontology and archaeology
☒	☐ Animals and other organisms
☒	☐ Clinical data
☒	☐ Dual use research of concern
☒	☐ Plants

Methods

n/a	Involved in the study
☒	☐ ChIP-seq
☒	☐ Flow cytometry
☒	☐ MRI-based neuroimaging

Plants

Seed stocks	*Report on the source of all seed stocks or other plant material used. If applicable, state the seed stock centre and catalogue number. If plant specimens were collected from the field, describe the collection location, date and sampling procedures.*
Novel plant genotypes	*Describe the methods by which all novel plant genotypes were produced. This includes those generated by transgenic approaches, gene editing, chemical/radiation-based mutagenesis and hybridization. For transgenic lines, describe the transformation method, the number of independent lines analyzed and the generation upon which experiments were performed. For gene-edited lines, describe the editor used, the endogenous sequence targeted for editing, the targeting guide RNA sequence (if applicable) and how the editor was applied.*
Authentication	*Describe any authentication procedures for each seed stock used or novel genotype generated. Describe any experiments used to assess the effect of a mutation and, where applicable, how potential secondary effects (e.g. second site T-DNA insertions, mosiacism, off-target gene editing) were examined.*
